# Expectation Learning for Stimulus Prediction Across Modalities Improves Unisensory Classification

**DOI:** 10.3389/frobt.2019.00137

**Published:** 2019-12-11

**Authors:** Pablo Barros, Manfred Eppe, German I. Parisi, Xun Liu, Stefan Wermter

**Affiliations:** ^1^Knowledge Technology, Department of Informatics, University of Hamburg, Hamburg, Germany; ^2^Department of Psychology, University of CAS, Beijing, China

**Keywords:** multisensory binding, deep learning, autoencoder, unsupervised learning, online learning

## Abstract

Expectation learning is a unsupervised learning process which uses multisensory bindings to enhance unisensory perception. For instance, as humans, we learn to associate a barking sound with the visual appearance of a dog, and we continuously fine-tune this association over time, as we learn, e.g., to associate high-pitched barking with small dogs. In this work, we address the problem of developing a computational model that addresses important properties of expectation learning, in particular focusing on the lack of explicit external supervision other than temporal co-occurrence. To this end, we present a novel hybrid neural model based on audio-visual autoencoders and a recurrent self-organizing network for multisensory bindings that facilitate stimulus reconstructions across different sensory modalities. We refer to this mechanism as stimulus prediction across modalities and demonstrate that the proposed model is capable of learning concept bindings by evaluating it on unisensory classification tasks for audio-visual stimuli using the 43,500 Youtube videos from the animal subset of the AudioSet corpus.

## 1. Introduction

Multisensory binding is one of the most important processes that humans use to understand their environment. By using different sensory mechanisms, we are able to collect and process distinct information streams from the same experience, which leads to a complex association learning. This mechanism allows us to improve the perception of individual stimuli (Frassinetti et al., 2002), solve contextual, spatial and temporal conflicts (Diaconescu et al., 2011), and progressively acquire and integrate novel information (Dorst and Cross, 2001).

There are different mechanisms involved in learning multisensory binding. One of the most important is the ability to process and understand unisensory information robustly (Macaluso, 2006). When the perception of individual stimuli has failed, the multisensory binding mechanism is affected by what is referred to as a multisensory illusion effect (Biocca et al., 2001). This effect creates artifacts via the binding mechanism which can influence the perception of other sensory stimuli (Driver, 1996; Mishra et al., 2007) and the formation of novel multisensory experiences (Spence and Driver, 2000). Our brain adapts to the multisensory illusion with a bottom-up selective mechanism (Soto-Faraco and Alsius, 2007) which shifts the attention resources over to the different sensing pipelines (Talsma et al., 2010).

An important aspect of multisensory bindings is known as the expectation effect (Yanagisawa, 2016). When perceiving an event, we compare it to other events we have experienced before, and make certain assumptions based on our experience. For instance, when seeing a cat, we expect it to meow and not to bark. This effect modulates our multisensory association in terms of top-down expectation. In consequence, when a cat barks at us, we assume that our perception is inconsistent, and that either the unisensory perception failed, or that the spatial or temporal attention was misleading. If we see barking cats repeatedly, we create a new concept of the species of a barking cat. For each of these scenarios, our brain adapts to the situation and we update our multisensory knowledge. This learning process, referred to as learning by expectation (Ashby and Vucovich, 2016), strongly suggests the role of unsupervised learning for multisensory binding, and leads to an adaptive mechanism for learning novel concepts (Ellingsen et al., 2016).

Despite its importance for human cognition and learning, there exists currently no functional computational model that is capable of modeling the multisensory binding and expectation learning effect in an unsupervised manner (see section 2). Such a model, however, would benefit from expectation learning as a mechanism to generate stimulus predictions across different sensory modalities. These cross-modal predictions potentially improve the robustness in perception and classification of unisensory stimuli through the binding of multisensory stimuli. This paper addresses the mentioned issues above by formalizing the following research questions:

Q.1 How can we build a computational model that allows for unsupervised learning of multisensory bindings?
Q.2 Can we adapt the expectation learning from humans to this model and use it to generate expected unisensory visual stimuli from auditory stimuli and vice versa?
Q.3 Can we exploit the generated expected stimuli to improve unisensory classification?
Q.4 How can we measure the quality of the learned multisensory bindings?

We address Q.1 in section 3, where we employ autoencoders to learn auditory and visual representations, which allows for unsupervised learning. As a novelty and innovative core mechanism to address continuity, we propose to link the autoencoders with a recurrent Grow-When-Required (GWR) neural network that changes its size as demanded, thus allowing for the unsupervised learning of multisensory bindings.

We address Q.2 in section 4 by demonstrating that the recurrent GWR network learns prototypes of multisensory bindings, which allows us to reconstruct auditory information from visual stimuli and vice versa. For example, when perceiving the sound of a cat, we expect the model to reconstruct the image of a cat, while when a dog enters a scene, the sound of the dog will be reconstructed. By extending the GWR association mechanism, we expect the model to be able to create concept-level bindings. Specifically, we hypothesize that by activating the neural units that represent prototypical concepts such as cats, dogs, and horses, the model will reconstruct prototypical auditory and visual stimuli in the absence of any sensory input. Our novel method is inspired by the multisensory imagery effect (Spence and Deroy, 2013), i.e., the ability of humans to create concepts from underspecified stimuli, and to use the abstract concepts to reconstruct unisensory information to enhance the overall perception.

We address Q.3 in sections 5 and 6, where we demonstrate the expectation learning effect can be used to improve the classification performance and hypothesize that our approach improves unisensory classification by reconstructing unisensory stimuli based on multisensory bindings.

To the best of our knowledge, there exists no standard benchmark to evaluate audio-visual bindings. Therefore, we propose an ablation study that includes a series of binding and classification experiments to address Q.4, and to assess the binding mechanism by measuring if and to what extent the expectation learning mechanism improves unisensory classification (see section 5). Herein, we employ the Youtube AudioSet corpus (Gemmeke et al., 2017) which contains human-labeled samples of Youtube videos based on the audio information. We select the animal subset of the corpus consisting of 44k samples to train the multisensory bindings in an unsupervised manner and exploit the multisensory bindings by using them to train a classifier for 24 different animal classes. We then employ the classifier to recognize absent stimuli, i.e., to recognize auditory stimuli when visual stimuli are present and vice versa.

To confirm our hypotheses, we summarize the results of our experiments in section 6 and show that the expectation learning improves the multisensory bindings in order to enhance the recognition of unisensory stimuli^1^. We analyze the results in section 7, providing evidence that correlates our network behavior with the multisensory imagery effect. Furthermore, we discuss the capabilities and limitations of our model. We conclude in section 8 that the expectation learning mechanism improves the quality of the multisensory association by providing a better unisensory classification.

## 2. Related Work

Most existing computational models for multisensory learning apply explicitly weighted connections, and the sensor information is integrated using early (Wei et al., 2010) or late (de Boer et al., 2016; Liu et al., 2016) fusion techniques. The weighted connections are usually tuned in a data-driven manner, whereby the data distribution directly affects the multisensory binding. Such existing methods have the drawback that they require supervision and that they are sensitive to the training data distribution when performing the multisensory integration. There exist computational models that are neurocognitively more accurate in the sense that they consider unisensory biases (Pouget et al., 2002; Rowland et al., 2007; Kayser and Shams, 2015). Such models, although similar to the brain's neural behavior, are usually not feasible to be used on real-world data, as they are mostly applied to simple stimuli scenarios, and do not scale well. There exist other complex models that implement attention mechanisms based on multisensory information, but the most recent focus in this area is on data-driven fusion models (Barros et al., 2017; Hori et al., 2017; Mortimer and Elliott, 2017). The introduction of expectation learning would give these models the ability to adapt better to novel situations and learn from their own errors in an online and continuous way.

Recent contributions build on data-driven learning for multisensory representations (Arandjelović and Zisserman, 2017a,b; Kim et al., 2018; Owens and Efros, 2018; Senocak et al., 2018). Such solutions employ transfer learning and attention mechanisms to improve unisensory recognition and localization. Although they provide solid results in these specific tasks, they rely on strongly labeled data points or have extensive training procedures that are not suitable for online learning. In particular, the work by Arandjelović and Zisserman (2017a) introduces a data-driven model for multisensory binding with bottom-up modulation for spatial attention. Their model uses the network's activity to spatially identify which part of an image a certain sound is related to. Although the model is data-driven, the authors claim that it learns real-world biasing on a multisensory description for unisensory retrieval by using a large amount of real-world training data. Their results show that the model can use multiple unisensory channels to compensate absent ones and identify congruent and incongruent stimuli.

A similar approach was presented by Zhou et al. (2017), who focus on audio generation. Their model relies on a sequence-to-sequence generator to associate audio events with visual information. The same generator is used to generate audio for newly presented video scenes. This requires an external teacher to identify congruent and incongruent stimuli which makes it impossible to be used in online learning scenarios.

All approaches that we summarized in this section depend on end-to-end learning that is not continuous. That is, the approaches cannot learn novel information without forgetting old information or extensively retraining the entire model. In the following, we discuss our GWR approach to address this issue.

## 3. Multisensory Temporal Binding

We divide the conceptual design of our model into two tasks: first, we propose a hybrid neural network that learns, in a fully unsupervised manner, to associate co-occurrent multisensory stimuli through a novel expectation learning mechanism. Once this network is trained, and the multisensory bindings are learned, we evaluate the learned bindings using a supervised classifier. This is necessary to guarantee that (1) our model learns in an unsupervised manner, without interference of giving labels, and (2) we provide a comparable objective metric for performance evaluation.

**Figure 1:**
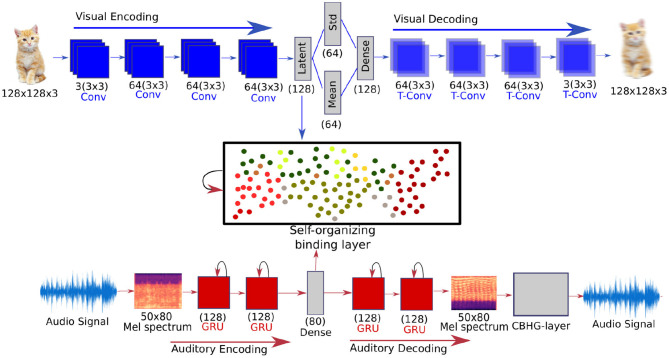
An overview of the proposed multisensory binding model with the audio/visual autoencoder structures and the recurrent self-organizing binding layer. The auditory autoencoder uses a CBHG network to reconstruct audio signals from the Mel Spectrum (Lee et al., 2017).

In our first task, we focus on multisensory binding learning. Our novel model learns based on the co-occurrence association enhanced through the reconstruction of expected stimuli. To reconstruct auditory and visual stimuli, we develop neural autoencoder networks for each of the unisensory channels. These networks encode high-dimensional data into a latent representation and reconstruct real-world audio-visual information. The binding between auditory and visual information is realized by means of a recurrent GWR network. The GWR is a self-organizing network that learns to create conceptual prototypes of data distributions in an unsupervised, incremental manner that allows for continuous learning. To address the temporal aspects of coincident binding, we extend the Gamma-GWR (Parisi and Wermter, 2017) which endows prototype neurons with a number of temporal contexts to learn the spatiotemporal structure of the data distribution. An overview of our multisensory binding model is illustrated in Figure 1.

In the second task, we train and evaluate a supervised classifier to evaluate the bindings. Note that the classifier does not participate in the learning process of the multisensory bindings; the learning of the bindings in the first tasks remains unsupervised, and no learning feedback is sent from it to the proposed model. Therefore, the learned bindings represent the multisensory co-occurrence and are not biased by supervision.

### 3.1. Visual Channel

To process high-level information by the visual channel, we drew inspiration from a variational autoencoder (VAE) (Kingma and Welling, 2013) which enforces the encoded latent variables to follow a Gaussian distribution.

In our experimental setup, the VAE produced better results when recognizing the animals from the AudioSet dataset when compared with normal convolution autoencoders. Recent studies demonstrate that the VAE learns how to extract useful information for image classification better than other unsupervised approaches on complex backgrounds (Li et al., 2017). Also, the embedding learned by the VAE showed to be more robust against noisy information and multi-view variance (Huang et al., 2018).

We assume that in our scenario, the improvement achieved with the VAE is due to the great variance on the image quality, perspective and resolution of the visual information of the images from the AudioSet dataset. Most likely the VAE learns to represent the important characteristics of the animals through the latent vector sampling instead of learning to reconstruct the entire encoded image. To train the VAE, we implemented a composite loss function based on the image reconstruction error and the Kullback-Leibler (KL) divergence between the encoded representation and the Gaussian unit. This composite loss function is important to enforce that the encoded representations learn general concepts of the animals instead of reconstructing input images from memorized parameters.

Our model receives as input a color image with a resolution of 128 × 128 × 3. The input data is processed by our encoding architecture which is composed of a series of four convolution layers, with a stride of 2 × 2, and kernel sizes of the dimension 3 × 3. The first convolution layer has three channels and the subsequent three layers have 64 filters. The latent representation starts with a fully connected layer with 128 units. The VAE computes the standard deviation and mean of this layer's output, generates a Gaussian distribution from it and samples an input for another fully connected hidden layer with 128 units, which is the final latent representation. The decoding layer has the same structure as our encoding layer but in the opposite direction and applying transpose convolutions.

We optimized the VAE using a tree-structured Parzen Estimator (TPE) (Bergstra et al., 2011) in order to minimize the visual reconstruction error. Table 1 exhibits all the important parameters used to train our vision channel. We used the ADAM optimizer with an adaptive learning rate.

**Table 1:** Training parameters of the vision channel.

**Parameter**	**Value**
Epochs	200
Batch size	32
Optimizer	ADAM
Initial learning rate	0.05
ADAM beta1	0.9
ADAM beta2	0.999

### 3.2. Auditory Channel

For the auditory channel, we implement a recurrent autoencoder based on Gated Recurrent Units (GRU) (Cho et al., 2014). Different from the vision channel, the auditory channel processes temporal information. As we have demonstrated in previous work, the auditory processing with autoencoders based on GRUs (Eppe et al., 2018a) obtained better representations than the ones with VAEs. We assume that this happens due to recurrent units allowing us to process and to reconstruct audio with better quality than when using non-recurrent layers since auditory signals are sequential, and each audio frame depends highly on previous contextual information (Eppe et al., 2018b).

As input and output of the auditory autoencoder, we compute a Mel spectrum which we generate from the raw waveform. To reconstruct the audio from the output Mel spectrum, we employ a convolutional bottleneck CBHG network model (Lee et al., 2017) which consists of a 1-D convolutional bank, a highway network and a bi-directional GRU layer. This network receives as input the Mel spectrum, and outputs a linear frequency spectrum which is then transformed into waveform using the Griffin Lim algorithm (Griffin and Jae Lim, 1984). This approach of transforming Mel coefficients into a linear spectrum and then into waveform achieved better audio synthesis quality than performing Griffin Lim on the Mel spectrum directly (Wang et al., 2017; Eppe et al., 2018a), and it improves the audio data of our expectation learning approach.

We performed hyperparameter optimization for the autoencoder and found that an audio spectrum window length of 50 ms, a window shift of 12.5 ms with 80 Mel coefficients and 1,000 linear frequencies yield best reconstruction results. We also found that 80 units for the dense bottleneck layer and two GRU layers with 128 units each for both the encoder and decoder network are sufficient for achieving a high audio quality. An additional number of Mel coefficients, GRU layers, and neural units did not significantly improve the reconstruction quality. The number of bottleneck units is important for the multisensory binding as it determines the number of connections between the binding layer and the audio encoder and decoder.

Similarly to the vision channel, we optimize the auditory channel using a tree-structured Parzen Estimator (TPE) (Bergstra et al., 2011) in order to minimize the auditory reconstruction error. Table 2 exhibits the important parameters used to train our auditory channel. We follow the same training procedure as the vision channel, and also used the ADAM optimizer with an adaptive learning rate.

**Table 2:** Training parameters of the auditory channel.

**Parameter**	**Value**
Epochs	250
Batch size	32
Optimizer	ADAM
Initial learning rate	0.01
ADAM beta1	0.9
ADAM beta2	0.999

### 3.3. Self-Organizing Temporal Binding

To learn coincident bindings between audio and visual stimuli, we use an unsupervised binding layer. An unsupervised learning strategy allows us to learn an online manner, where the bindings are created based on the data distribution. Also, excluding an external teaching signal allows the bindings to learn how to best represent the co-incident multisensory stimuli. In this regard, Growing-When-Required (GWR) networks have been recently explored as continual learning mechanism (Parisi et al., 2019). Their capability to grow and shrink, adding and removing neurons while they are learning, made them experts on avoiding catastrophic forgetting (Soltoggio et al., 2018). Such networks, however, are experts on learning topological relations between the input data. To be able to process co-incident multisensory stimuli, we propose here the implementation of a recurrent GWR layer which receives as input the latent representations of our visual and auditory channels which are processed coincidentally, and learn how to create prototype neurons which represent the multisensory binding.

To synchronize the two data streams, we resample video and audio streams to a temporal resolution of 20 frames per second, i.e., each video frame is associated with 12.5 ms of auditory information. In contrast to traditional self-organizing models with winner-takes-all dynamics for the processing of spatial patterns, the Gamma-GWR (Parisi and Wermter, 2017) computes the winner neuron taking into account the activity of the network for the current input and a temporal context. Each neuron of the map consists of a weight vector **w**_*j*_ and a number *K* of context descriptors ${\text{c}}_{j}^{k}$ (with ${\text{w}}_{j},{\text{c}}_{j}^{k}\in {\mathbb{R}}^{n}$). As a result, recurrent neurons in the map will encode prototype sequence-selective snapshots of the input. Given a set of *N* neurons, the best-matching unit (BMU), **b**, with respect to the input **x**(*t*) ∈ ℝ^*n*^ is computed as:

(1) $$\begin{array}{r} b=\arg\underset{j\in N}{\min}\left(\alpha_{0}∥\text{x}\left(t\right)-{\text{w}}_{j}{∥}^{2}+\overset{K}{\underset{k=1}{\sum }}\;\alpha_{k}∥{\text{C}}_{k}\left(t\right)-{\text{c}}_{j,k}{∥}^{2}\right), \end{array}$$

(2) $$\begin{array}{r} {\text{C}}_{k}\left(t\right)=\beta \cdot {\text{w}}_{I\left(t-1\right)}+\left(1-\beta \right)\cdot {\text{c}}_{I\left(t-1\right),k-1}, \end{array}$$

where α_*i*_ and β∈(0;1) are constant values that modulate the influence of the current input with respect to previous neural activity, **w**_*I*_(*t* − 1) is the weight of the winner neuron at *t* − 1, and ${\text{C}}_{k}\in {\mathbb{R}}^{n}$ is the global context of the network (**C**_*k*_(*t*_0_) = 0).

New connections are created between the BMU and the second BMU for any given input. When a BMU is computed, all the neurons the BMU is connected to are referred to as its topological neighbors. Each neuron is equipped with a habituation counter *h*_*i*_ ∈ [0, 1] expressing how frequently it has fired based on a simplified model of how the efficacy of a habituating synapse reduces over time. In the Gamma-GWR, the habituation rule is given by Δ*h*_*i*_ = τ_*i*_·κ·(1 − *h*_*i*_) − τ_*i*_, where κ and τ_*i*_ are constants that control the decreasing behavior of the habituation counter (Marsland et al., 2002). We say that a neuron is habituated, if its habituation counter *h*_*i*_ is smaller than a given habituation threshold *h*_*T*_. The network is initialized with two neurons and, at each learning iteration, it inserts a new neuron whenever the activity of the network *a*(*t*) of a habituated neuron is smaller than a given threshold *a*_*T*_, i.e., a new neuron *r* is created if *a*(*t*) < *a*_*T*_ and *h*_*b*_ < *h*_*T*_. The training of the neurons is carried out by adapting the BMU *b* and its topological neurons *n* according to:

(3) $$\begin{array}{r} \Delta {\text{w}}_{i}=\epsilon_{i}\cdot h_{i}\cdot \left(\text{x}\left(t\right)-{\text{w}}_{i}\right), \end{array}$$

(4) $$\begin{array}{r} \Delta {\text{c}}_{k,i}=\epsilon_{i}\cdot h_{i}\cdot \left({\text{C}}_{k}\left(t\right)-{\text{c}}_{k,i}\right), \end{array}$$

where ϵ_*i*_ is a constant learning rate. The learning process of the Gamma-GWR is unsupervised and driven by bottom-up sensory observations, thereby either allocating new neurons or adapting existing ones in response to novel input. In this way, fine-grained multisensory representations can be acquired and fine-tuned through experience.

As an extension of the Gamma-GWR, we implement temporal connections for the purpose of predicting future frames from an onset frame. The temporal connections are implemented as sequence-selective synaptic links that are incremented between those two neurons that are consecutively activated. When the two neurons *i* and *j* are activated at time *t* − 1 and *t*, respectively, their synaptic link *P*_(*i, j*)_ is strengthened. Thus, at each learning iteration, we set Δ*P*_(*I* − 1, *b*)_ = 1, where *I*−1 and *b* are the indexes of the BMUs at time *t* − 1 and *t*, respectively. As a result, for each neuron *i* ∈ *N*, we can retrieve the next neuron *v* of a prototype sequence by selecting

(5) $$\begin{array}{r} v=\arg\underset{j\in N\backslash i}{\max}P_{\left(i,j\right)}. \end{array}$$

This approach results in the learning of trajectories of neural activations that can be reconstructed in the absence of sensory input. We also optimized the parameters of the Gamma-GWR using a tree-structured Parzen Estimator (TPE) (Bergstra et al., 2011) minimizing the network's quantization error. Table 3 exhibits the parameters used to train our Gamma Growing-When-Required (Gamma-GWR) network. We use a small insertion threshold, which helps the network to maintain a limited number of neurons, reinforcing the generation of highly abstract clusters.

**Table 3:** Training parameters of the self-organizing temporal binding layer.

**Parameter**	**Value**
Epochs	50
Insertion threshold	0.01
Context size	4
Initial Gamma Weights	0.64391426, 0.23688282, 0.08714432, 0.0320586
β_*b*_	0.5
ϵ_*b*_	0.2
ϵ_*n*_	0.003

### 3.4. Supervised Classifiers

The supervised classifiers were implemented to generate an objective performance metric of the unsupervised learning mechanism. In this regard, they are trained in a separated training step which does not influence the multisensory binding learning. We provide two classifiers, one for vision and one for audio, to measure the unisensory recognition capabilities of the learned multisensory bindings.

Each classifier receives as input the audio or visual part of the BMU, depending on which unisensory stimuli we want to classify, of the GWR which represents the perceived stimuli. Each classifier is composed of a dense layer with 128 units and an output softmax layer. Similarly to the autoencoders and the GWR, we optimized the classifiers to maximize the recognition accuracy using a tree-structured Parzen Estimator (TPE) (Bergstra et al., 2011) and use the optimal parameters through all of our experiments (see Table 3). An overview of the proposed multisensory binding model with the audio/visual autoencoder structures and the recurrent self-organizing binding layer. The auditory autoencoder uses a CBHG network to reconstruct audio signals from the Mel Spectrum.

## 4. Expectation Learning

As the self-organizing layer is updated in an unsupervised Hebbian manner, it learns to associate audio-visual stimuli online. This implies that the binding process is entirely co-occurrent-driven, without the necessity of external supervision other than temporal co-occurrence. More specifically, after finding the BMU related to a unimodal perceived stimulus, the associated absent stimuli will be reconstructed based on the prototypical concept that this neuron learned. This is possible because each neuron in the self-organizing layer processes the union of the auditory and visual encodings at training time, where both signals are provided.

The reconstruction and expectation learning capability is the basis for our novel proposal of a expectation learning mechanism for the self-organizing layer. First, we pre-train our self-organizing binding to generate prototype neurons with strong audio-visual encodings. This allows the model to learn a prior association between auditory and visual concepts. Second, after the network has learned these associations, we use unseen data points to fine-tune the bindings with the expectation learning through the update of the learned associations using the reconstructed stimuli.

The network encodes a visual or auditory stimulus (*s*), and computes the BMU (*b*_*av*_) using only the associated auditory or visual weights as follows:

(6) $$\begin{array}{r} b_{av}=\arg\underset{j\in N}{\min}\left(\alpha_{0}∥\text{s}\left(t\right)-{\tilde{\text{w}}}_{j}^{s}{∥}^{2}+\overset{K}{\underset{k=1}{\sum }}\;\alpha_{k}∥{\tilde{\text{C}}}_{k}^{s}\left(t\right)-{\tilde{\text{c}}}_{j,k}{∥}^{2}\right), \end{array}$$

where ${\tilde{\text{w}}}_{j}^{s}$ represents the audio or visual representation encoded on the neuron's weights. In this case, the global context of the network at any time step (${\tilde{\text{C}}}_{k}^{s}\left(t\right)$) is represented by the stimulus encoding; the same happens with the BMU context (${\tilde{\text{c}}}_{j,k}$). We then use the auditory and vision parts of the multisensory representation stored on *b*_*av*_ to reconstruct the auditory (*a*′) and visual (*v*′) information using the specific channel decoding *D*_*v*_ for vision and *D*_*a*_ for audio:

(7) $$\begin{array}{l} a^{\prime }=D_{a}\left(b_{a}\right),\\ s^{\prime }=D_{v}\left(b_{v}\right). \end{array}$$

When the model processes the perceived auditory and visual signals, it creates two extra pairs of multisensory stimuli by combining the perceived auditory and visual ones with the reconstructed auditory and visual stimuli. We bind the encoded information of the reconstructed audio-visual information to the originally perceived stimuli and re-train the self-organizing layer with the new pairs. By pairing the perceived and the reconstructed stimuli representations, we enforce the self-organizing layer to learn general concepts, and not specific instances of the animals. In consequence, animals which sound similar will be paired together, and connections of coincident stimuli will be learned with relatively small amounts of training data. Inconsistencies will cause the model to pair different audio-visual stimuli, thus creating new prototype neurons, but these will be forgotten quickly by the self-organizing layer as they occur less frequently.

## 5. Experimental Setup

Our goal is to evaluate the performance of the model to reconstruct audio/visual stimuli based on unimodal perception, and to evaluate the conceptual relations learned by the network. Although there exist several datasets with multimodal information, the animal subset of the AudioSet corpus^2^ (Gemmeke et al., 2017) presents a unique advantage for our evaluation: It contains natural scenarios with different levels of conceptual binding, including broader prototype associations like images of cats linked to meowing, but also more fine-grained associations like high-pitched barking linked to small dogs.

Each video in the dataset has a duration of 10 s and it is possible that, e.g., there is both a cat and a dog present in the video. As there are no standard published results of this specific task for the AudioSet corpus, we run a series of baseline recognition experiments that serve as the main comparison to measure our model's performance. To obtain a precise measure of the contribution of the expectation learning, we decide to cluster some overlapping classes and use 16 single labels, one per video: Cats (“Cat” + “Meow” + “Purr”), Dogs (“Bark” + “Dog” + “Howl”), Pigs (“Oink” + “Pig”), Cows (“Moo” + “Cattle, bovinae”), Owls (“Owl” + “Coo”), Birds, Goats, Bee (“Bee, wasp, etc.”), Chickens (“Chicken, rooster”), Ducks (“Duck”), Pidgeons (“Pidgeon, dove”), Crows (“Crow”), Horses (“Horse”), Frogs (“Frogs”), Flies (“Fly, housefly”), Lions (“Roaring cats (lions, tigers)”). We use the unbalanced training subset consisting of approximately 43,500 videos to train our model and evaluated it with the test subset consisting of approximately 20,000 videos. The labels of this dataset are crowdsourced based on the video descriptions.

We perform two sets of experiments: one to evaluate the contribution of the expectation learning to the multisensory binding and one to compare the performance of our model with currently successful deep learning models for unisensory recognition.

The first set of experiments is divided into three steps. In EXP 1.1, we train the multisensory bindings of the GWR using half of the training subset in order to guarantee that the model learns strong audio-visual prior bindings. In EXP 1.2, we continue the training of the EXP 1.1 network using the other half of the training subset. This experiment serves as a baseline for learning bindings without expectation and as a main comparison point for the contribution of the expectation learning mechanism. Finally, in EXP 1.3, we repeat the continuation of the training of the EXP 1.1 network with the other half of the training subset but now using the expectation learning mechanism when creating the GWR associations.

To evaluate the performance contribution of each of our experimental steps on the association learning we use the implemented supervised classifiers for each of the channels (auditory and visual). To evaluate the capability of the model to learn meaningful associations, we always classify an absent stimulus, i.e., when perceiving an auditory stimulus, the network uses the associated visual stimulus as input to the classifier and vice versa. This means that, when perceiving 50ms of audio, we have an associated representation of 4 frames and vice versa. As the videos from the AudioSet dataset have a length of 10s, we use a simple voting scheme to obtain the final label. For every 50 ms of audio and every 4 frames per video, we produce one label and after having all the labels for a 10 s video, we select the one which appears most often.

Our second set of experiments is designed to evaluate how our proposed model compares with deep learning networks for auditory and visual stimuli recognition. In EXP 2.1, we compare our model with the Inception V3 network (Ioffe and Szegedy, 2015) for the visual stimuli, and in EXP 2.2 with the SoundNet (Aytar et al., 2016) for the auditory stimuli. These two models present competitive results on different audio-visual recognition tasks (Jansen et al., 2018; Jiang et al., 2018; Kiros et al., 2018; Kumar et al., 2018). For all experiments, we trained the models 10 times and determined the mean accuracy and standard deviation for each modality. We used the same 10% of the training subset as a validation set for each experiment, and used an early stopping mechanism based on the accuracy of the validation subset to prevent overfitting.

## 6. Results

Our final results are depicted in Table 4. Our first experiment, EXP 1.1, demonstrates that training the model with half of the data, to create strong binding associations, is enough to obtain a baseline performance. Continuing to train the model using standard GWR associations (EXP 1.2) shows the expected improvement, i.e., an 8% gain in the recognition accuracy for audio and more than 17% of accuracy gain for vision when compared to EXP 1.1. The results of EXP 1.3 show that the expectation mechanism improves the recognition of unisensory stimuli, when compared to EXP 1.2. We obtained an improvement of more than 4% on audio and 3% on vision.

**Table 4:** Mean accuracy, in percentage, and standard deviation of our experiments.

**Exp**.	**Model**	**Audio**	**Vision**
*EXP 1.1*	Prior binding association	58.5 (3.1)	69.0 (3.9)
*EXP 1.2*	Without expectation	66.4 (2.4)	86.8 (3.2)
*EXP 1.3*	With expectation	70.8 (3.2)	89.8 (1.9)
*EXP 2.1*	Inception V3 (Ioffe and Szegedy, 2015)	–	89.4 (1.3)
*EXP 2.2*	SoundNet (Aytar et al., 2016)	68.5 (2.4)	–

The performance of the network follows the general behavior of other models to recognize vision stimuli better than auditory stimuli. This effect is demonstrated by the results of the Inception-V3 (EXP 2.1) and the SoundNet (EXP 2.2) models. This is probably due to the dataset presenting challenging audio stimuli with much background noise.

When compared with Inception-V3 (EXP 2.1) and SoundNet (EXP 2.2), our expectation model (EXP 1.3) presents better auditory recognition, and slightly better vision recognition performance. The auditory stimulus is more affected, as it presents much more noisy information. In the latter case, the network relies more on the visual stimuli and creates neurons with strong visual encoding. This effect is represented by creating neurons with similar visual encoding associated with the auditory encoding. When training with expectation learning, the network creates an average of 5,400 neurons, while when training without the expectation, it creates 4,000 neurons.

**Figure 2:**
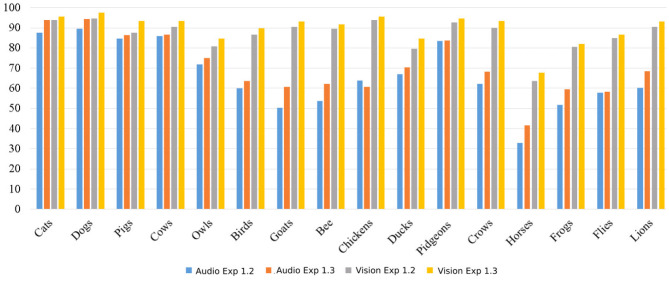
Mean accuracy per class, in percentage, of the reconstructed absent stimuli. We compare audio and visual reconstruction with the results when training the network with all the samples of the training set.

The latent representations from the auditory and visual channels encode different characteristics of the stimulus and are then connected by our self-organizing layer. The expectation learning enforces the generation of robust bindings, especially for distinct animals. For example, the network eventually created specific neurons for cats and dogs and shared neurons for chickens and ducks. This explains the improvement of the recognition of the reconstructed stimuli of easily separable animals, as illustrated by the differences between the accuracy differences of the cats and horses categories in Figure 2.

This behavior can be easily observed when comparing the mean accuracy and standard deviation per class of our baseline experiments (SoundNet and Inception V3) with the detailed accuracy per class obtained by our expectation learning model (see Table 5). Animal classes which more distinct between each other presents a better accuracy and standard deviation.

**Table 5:** Mean accuracy, in percentage, and standard deviation of our experiments per classification class.

**Animal class**	**Audio**			**Vision**		
**–**	**SoundNet**	**Without**	**With**	**Inception**	**Without**	**With**
		**Expectation**	**Expectation**	**V3**	**Expectation**	**Expectation**
Cats	90.2 (3.2)	87.6 (3.2)	93.8 (2.1)	94.8 (2.4)	93.8 (1.9)	95.6 (2.1)
Dogs	92.5 (4.1)	89.5 (3.6)	94.4 (2.9)	96.7 (2.5)	94.6 (2.2)	97.5 (1.8)
Pigs	80.7 (3.7)	84.6 (3.2)	86.5 (3.7)	95.6 (3.4)	87.5 (1.4)	93.4 (1.7)
Cows	83.8 (3.5)	85.9 (4.1)	86.7 (2.7)	94.8 (1.7)	90.4 (1.6)	93.4 (2.8)
Owls	71.8 (1.4)	71.8 (3.7)	74.9 (2.9)	87.8 (1.0)	80.7 (1.8)	84.7 (1.9)
Birds	62.7 (2.2)	60.1 (2.6)	63.7 (1.9)	90.6 (3.6)	86.7 (4.7)	89.7 (3.7)
Goats	60.2 (3.9)	50.2 (1.6)	60.7 (3.7)	95.8 (2.1)	90.4 (2.8)	93.2 (1.9)
Bee	63.1 (1.1)	53.7 (2.7)	62.1 (3.9)	91.2 (4.7)	89.5 (2.7)	91.7 (3.1)
Chickens	59.8 (3.0)	63.8 (1.9)	60.7 (2.1)	85.1 (1.7)	93.8 (1.7)	95.7 (1.9)
Ducks	68.7 (4.1)	66.9 (1.9)	70.5 (2.8)	96.8 (2.3)	79.5 (1.6)	84.6 (2.9)
Pidgeons	76.8 (2.6)	83.6 (4.7)	83.8 (2.6)	92.5 (3.1)	92.6 (2.7)	94.7 (2.9)
Crows	67.9 (1.8)	62.1 (1.9)	68.3 (2.2)	91.3 (2.7)	90.1 (2.0)	93.4 (2.8)
Horses	43.6 (3.7)	32.8 (2.6)	41.6 (3.9)	69.8 (4.1)	63.7 (3.1)	67.8 (1.8)
Frogs	57.8 (1.4)	51.8 (3.7)	59.4 (2.7)	79.8 (2.5)	80.6 (2.7)	82.1 (3.4)
Flies	53.1 (1.3)	57.8 (3.0)	58.3 (2.5)	89.8 (1.9)	84.9 (1.6)	86.7 (2.6)
Lions	63.5 (3.4)	60.3 (2.9)	68.5 (2.6)	94.5 (2.5)	90.4 (2.4)	93.2 (3.8)

## 7. Discussion

As the self-organizing layer is updated in an unsupervised manner, it learns to associate audio-visual stimuli online. Moreover, by activating the BMU related to a specific perceived stimulus, the associated absent stimulus can be reconstructed based on the concept that this neuron learned. However, the reconstructed data is, of course, not identical to the original data. For example, when processing an image of a dog, the network will reconstruct an appropriate barking sound, but not exactly the sound that this specific dog would make. This mimics precisely the multisensory imagery effect (Spence and Deroy, 2013) of humans, who tend to simplify and cluster absent stimuli when asked to reconstruct them. For example, every time one sees a small yellow bird, the person will expect it to sound very similar to the ones she/he has seen before. This is an important effect that helps our model to reconstruct animal concepts instead of specific instances.

To provide an indication of this effect, and as an additional indicator for multisensory concept formation, we performed an additional overlapping analysis to estimate how well the model is binding and clustering audio-visual information. To this end, we first train the model with the expectation learning mechanism and then we classify every single neuron of the GWR using both audio and visual classifiers which generate two labels for each neuron: one for auditory and one for visual information. The total overlap between visual and auditory labels for each prototype neuron in our self-organizing layer is 93%, suggesting that our prototype neurons are very concise when storing audio-visual information. Performing the same experiment on the network training without the expectation mechanism gave us an overlap of 85% for the neurons.

**Figure 3:**
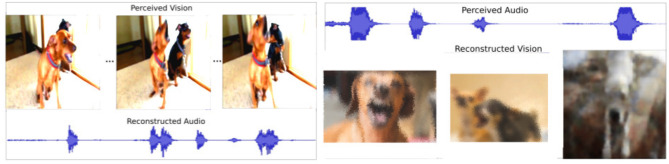
Example of the reconstruction output. The left image displays the audio reconstruction when the visual stimulus is perceived. The right image displays the vision reconstruction when the audio stimulus is perceived.

Another effect that we investigate is multisensory correspondence (Spence and Driver, 2000). The effect causes humans not only to associate dogs with barking but also, more specifically, small dogs with high-pitched barking. The associations between the stimuli are continuously reinforced when perceptive stimuli are experienced. We observed this effect in some examples where the variety of animals was higher, such as dogs. We illustrate one of these examples in Figure 3. The figure depicts the reconstruction of visual information based on an auditory stimulus of different dogs barking. A high-pitched barking generates images related to a small dog. Furthermore, when the simultaneous barking of more than one dog is processed, the network generates an image of several dogs. We expect this effect to become more visible with larger datasets that contain more diverse samples.

The cognitive plausibility of our approach is underpinned by an important limitation: Both multisensory imagery and multisensory correspondence only occurs when both auditory and visual stimuli can be understood and represented as a simplified concept. This also holds for human cognition: For example, humans cannot reconstruct precisely the characteristics of how the voice of a person will sound when reading a text. Our experiments demonstrate that our model learns to associate high-level animal concepts, and even multisensory correspondences, but could not be applied to reconstruct information that demands a much higher precision, i.e., person identification.

## 8. Conclusion

Multisensory binding is a crucial aspect of how humans understand the world. Consequently, the development of computational systems able to adapt this aspect into information processing is important to many research fields. An extensive number of models has been proposed that incorporate different aspects of multisensory binding. However, our approach combines several novelties. It combines a Grow-When-Required (GWR) network with convolutional autoencoders to realize unsupervised expectation learning. In addition, we propose to exploit expectation learning by reconstructing stimuli that can be used as additional training data to generate a significant positive effect on perceptive tasks like classification. We, therefore, provide a novel proof of concept for a data augmentation mechanism to improve the accuracy and performance of unimodal classification methods.

An interesting future research direction is to also address spatial expectation, because this would provide a complementary component to integrate contextual, temporal, and spatial correspondence. Realizing the transfer of learned multisensory bindings is another unexplored research area that we plan to investigate as a follow-up to this work. To model the multisensory characteristics of the classification, in particular aspects regarding multisensory conflict resolution and fusion would be an interesting next step as well.

## Data Availability Statement

Publicly available datasets were analyzed in this study. This data can be found here: https://research.google.com/audioset/.

## Author Contributions

All authors listed have made a substantial, direct and intellectual contribution to the work, and approved it for publication.

### Conflict of Interest

The authors declare that the research was conducted in the absence of any commercial or financial relationships that could be construed as a potential conflict of interest.

## Footnotes

^1^Note that our approach is different from multimodal classification where multiple sensory modalities are necessary to recognize the class of a stimulus.

^2^https://research.google.com/audioset/

## References

[B1] Arandjelović R. Zisserman A. (2017a). Look, listen and learn, in IEEE International Conference on Computer Vision (ICCV) (Venice), 609–617. 10.1109/ICCV.2017.73

[B2] Arandjelović R. Zisserman A. (2017b). Objects that sound. arXiv:1712.06651.

[B3] Ashby F. G. Vucovich L. E. (2016). The role of feedback contingency in perceptual category learning. J. Exp. Psychol. Learn. Mem. Cogn. 42:1731. 10.1037/xlm0000277 27149393 PMC5097011

[B4] Aytar Y. Vondrick C. Torralba A. (2016). Soundnet: learning sound representations from unlabeled video, in Conference on Neural Information Processing Systems (NIPS) (Barcelona), 892–900. 10.1109/CVPR.2016.18

[B5] Barros P. Parisi G. I. Weber C. Wermter S. (2017). Emotion-modulated attention improves expression recognition: a deep learning model. Neurocomputing 253, 104–114. 10.1016/j.neucom.2017.01.096

[B6] Bergstra J. S. Bardenet R. Bengio Y. Kégl B. (2011). Algorithms for hyper-parameter optimization, in Conference on Neural Information Processing Systems (NIPS) (Granada), 2546–2554.

[B7] Biocca F. Kim J. Choi Y. (2001). Visual touch in virtual environments: an exploratory study of presence, multimodal interfaces, and cross-modal sensory illusions. Presence 10, 247–265. 10.1162/105474601300343595

[B8] Cho K. van Merrienboer B. Gulcehre C. Bahdanau D. Bougares F. Schwenk H. (2014). Learning phrase representations using RNN encoder-decoder for statistical machine translation, in Conference on Empirical Methods in Natural Language Processing (EMNLP) (Doha), 1724–1734.

[B9] de Boer M. H. T. Schutte K. Zhang H. Lu Y.-J. Ngo C.-W. Kraaij W. (2016). Blind late fusion in multimedia event retrieval. Int. J. Multimedia Informat. Retrieval 5, 203–217. 10.1007/s13735-016-0112-9

[B10] Diaconescu A. O. Alain C. McIntosh A. R. (2011). The co-occurrence of multisensory facilitation and cross-modal conflict in the human brain. J. Neurophysiol. 106, 2896–2909. 10.1152/jn.00303.2011 21880944

[B11] Dorst K. Cross N. (2001). Creativity in the design process: co-evolution of problem–solution. Design Stud. 22, 425–437. 10.1016/S0142-694X(01)00009-6

[B12] Driver J. (1996). Enhancement of selective listening by illusory mislocation of speech sounds due to lip-reading. Nature 381:66. 10.1038/381066a0 8609989

[B13] Ellingsen D.-M. Leknes S. Løseth G. Wessberg J. Olausson H. (2016). The neurobiology shaping affective touch: expectation, motivation, and meaning in the multisensory context. Front. Pychol. 6:1986. 10.3389/fpsyg.2015.01986 26779092 PMC4701942

[B14] Eppe M. Alpay T. Wermter S. (2018a). Towards end-to-end raw audio music synthesis, in International Conference on Artificial Neural Networks (ICANN) (Rhodes), 137–146. 10.1007/978-3-030-01424-7_14

[B15] Eppe M. Kerzel M. Strahl E. Wermter S. (2018b). Deep neural object analysis by interactive auditory exploration with a humanoid robot, in IEEE International Conference on Intelligent Robots and Systems (IROS) (Madrid: IEEE Publishing), 284–289.

[B16] Frassinetti F. Bolognini N. Làdavas E. (2002). Enhancement of visual perception by crossmodal visuo-auditory interaction. Exp. Brain Res. 147, 332–343. 10.1007/s00221-002-1262-y 12428141

[B17] Gemmeke J. F. Ellis D. P. W. Freedman D. Jansen A. Lawrence W. Moore R. C. (2017). Audio set: an ontology and human-labeled dataset for audio events, in IEEE International Conference on Acoustics, Speech and Signal Processing (ICASSP) (New Orleans, LA).

[B18] Griffin D. Jae Lim (1984). Signal estimation from modified short-time Fourier transform. IEEE Trans. Acoust. Speech Sig. Process. 32, 236–243. 10.1109/TASSP.1984.1164317

[B19] Hori C. Hori T. Lee T.-Y. Zhang Z. Harsham B. Hershey J. R. (2017). Attention-based multimodal fusion for video description, in IEEE International Conference on Computer Vision (ICCV)omputer Vision (ICCV) (Venice), 4203–4212.

[B20] Huang F. Zhang X. Li C. Li Z. He Y. Zhao Z. (2018). Multimodal network embedding via attention based multi-view variational autoencoder, in ACM International Conference on Multimedia Retrieval (Dublin), 108–116.

[B21] Ioffe S. Szegedy C. (2015). Batch normalization: accelerating deep network training by reducing internal covariate shift, in International Conference on Machine Learning (ICML) (Lille), 448–456.

[B22] Jansen A. Plakal M. Pandya R. Ellis D. P. W. Hershey S. Liu J. (2018). Unsupervised learning of semantic audio representations, in IEEE International Conference on Acoustics, Speech and Signal Processing (ICASSP) (Calgary, AB), 126–130.

[B23] Jiang L. Zhou Z. Leung T. Li L.-J. Fei-Fei L. (2018). MentorNet: learning data-driven curriculum for very deep neural networks on corrupted labels, in International Conference on Machine Learning (ICML) (Stockholm), 2309–2318.

[B24] Kayser C. Shams L. (2015). Multisensory causal inference in the brain. PLoS Biol. 13:e1002075. 10.1371/journal.pbio.1002075 25710476 PMC4339834

[B25] Kim C. Shin H. V. Oh T.-H. Kaspar A. Elgharib M. Matusik W. (2018). On learning associations of faces and voices. arXiv:1805.05553.

[B26] Kingma D. P. Welling M. (2013). Auto-encoding Variational Bayes. Technical report.

[B27] Kiros J. Chan W. Hinton G. (2018). Illustrative language understanding: large-scale visual grounding with image search, in Annual Meeting of the Association for Computational Linguistics (ACL) (Melbourne, VIC) 1, 922–933.

[B28] Kumar A. Khadkevich M. Fügen C. (2018). Knowledge transfer from weakly labeled audio using convolutional neural network for sound events and scenes, in IEEE International Conference on Acoustics, Speech and Signal Processing (ICASSP) (Calgary, AB), 326–330.

[B29] Lee J. Cho K. Hofmann T. (2017). Fully character-level neural machine translation without explicit segmentation. Trans. Assoc. Comput. Linguist. 5, 365–378. 10.1162/tacl_a_00067

[B30] Li H. Wang H. Yang Z. Odagaki M. (2017). Variation autoencoder based network representation learning for classification, in ACL Student Research Workshop (Vancouver, BC), 56–61.

[B31] Liu J.-C. Chiang C.-Y. Chen S. (2016). Image-based plant recognition by fusion of multimodal information, in International Conference on Innovative Mobile and Internet Services in Ubiquitous Computing (IMIS) (Fukuoka), 5–11.

[B32] Macaluso E. (2006). Multisensory processing in sensory-specific cortical areas. Neuroscientist 12, 327–338. 10.1177/1073858406287908 16840709

[B33] Marsland S. Shapiro J. Nehmzow U. (2002). A self-organising network that grows when required. Neural Netw. 15, 1041–1058. 10.1016/S0893-6080(02)00078-3 12416693

[B34] Mishra J. Martinez A. Sejnowski T. J. Hillyard S. A. (2007). Early cross-modal interactions in auditory and visual cortex underlie a sound-induced visual illusion. J. Neurosci. 27, 4120–4131. 10.1523/JNEUROSCI.4912-06.2007 17428990 PMC2905511

[B35] Mortimer B. J. P. Elliott L. R. (2017). Information transfer within human robot teams: multimodal attention management in human-robot interaction, in IEEE Conference on Cognitive and Computational Aspects of Situation Management (CogSIMA) (Savahna, GA), 1–3.

[B36] Owens A. Efros A. A. (2018). Audio-visual scene analysis with self-supervised multisensory features. arXiv:1804.03641. 10.1007/978-3-030-01231-1_39

[B37] Parisi G. I. Kemker R. Part J. L. Kanan C. Wermter S. (2019). Continual lifelong learning with neural networks: a review. Neural Netw. 113, 54–71. 10.1016/j.neunet.2019.01.012 30780045

[B38] Parisi G. I. Wermter S. (2017). Lifelong learning of action representations with deep neural self-organization, in AAAI Spring Symposium (Palo Alto, CL), 608–612.

[B39] Pouget A. Deneve S. Duhamel J.-R. (2002). A computational perspective on the neural basis of multisensory spatial representations. Nat. Rev. Neurosci. 3:741. 10.1038/nrn914 12209122

[B40] Rowland B. A. Stanford T. R. Stein B. E. (2007). A model of the neural mechanisms underlying multisensory integration in the superior colliculus. Perception 36, 1431–1443. 10.1068/p5842 18265826

[B41] Senocak A. Oh T.-H. Kim J. Yang M.-H. Kweon I. S. (2018). Learning to localize sound source in visual scenes, in IEEE Conference on Computer Vision and Pattern Recognition (Salt Lake City, UT), 4358–4366. 10.1109/CVPR.2018.00458

[B42] Soltoggio A. Stanley K. O. Risi S. (2018). Born to learn: the inspiration, progress, and future of evolved plastic artificial neural networks. Neural Netw. 108, 48–67. 10.1016/j.neunet.2018.07.013 30142505

[B43] Soto-Faraco S. Alsius A. (2007). Conscious access to the unisensory components of a cross-modal illusion. Neuroreport 18, 347–350. 10.1097/WNR.0b013e32801776f9 17435600

[B44] Spence C. Deroy O. (2013). Crossmodal mental imagery, in Multisensory Imagery, ed Lacey S. Lawson R. (New York, NY: Springer), 157–183.

[B45] Spence C. Driver J. (2000). Attracting attention to the illusory location of a sound: reflexive crossmodal orienting and ventriloquism. Neuroreport 11, 2057–2061. 10.1097/00001756-200006260-00049 10884070

[B46] Talsma D. Senkowski D. Soto-Faraco S. Woldorff M. G. (2010). The multifaceted interplay between attention and multisensory integration. Trends Cogn. Sci. 14, 400–410. 10.1016/j.tics.2010.06.008 20675182 PMC3306770

[B47] Wang Y. Skerry-Ryan R. J. Stanton D. Wu Y. Weiss R. J. Jaitly N. (2017). Tacotron: Towards End-to-End Speech Synthesis. Technical report, Google, Inc.

[B48] Wei S. Zhao Y. Zhu Z. Liu N. (2010). Multimodal fusion for video search reranking. IEEE Trans. Knowl. Data Eng. 22, 1191–1199. 10.1109/TKDE.2009.145

[B49] Yanagisawa H. (2016). Expectation effect theory and its modeling, in Emotional Engineering, Vol. 4, ed Fukuda S. (Cham: Springer), 199–211.

[B50] Zhou Y. Wang Z. Fang C. Bui T. Berg T. L. (2017). Visual to sound: generating natural sound for videos in the wild. arXiv:1712.01393. 10.1109/CVPR.2018.00374

